# Stability and change of psychopathology symptoms among youth with chronic physical illness: A latent transition analysis

**DOI:** 10.1177/13591045251356430

**Published:** 2025-06-26

**Authors:** Megan Dol, Dillon Browne, Christopher M Perlman, Mark A Ferro

**Affiliations:** 1 School of Public Health Sciences, 8430 University of Waterloo, Canada; 2 Department of Psychology, 8430 University of Waterloo, Canada

**Keywords:** Mental disorders, child, adolescent, latent profile analysis, latent transition analysis

## Abstract

**Background:**

This study examined whether youth transition between different mental health symptom profiles over time, and what factors predict these transitions. Understanding the stability and change in psychopathology helps discern whether certain behaviours are temporary or signs of persistent problems.

**Methods:**

Data were drawn from a longitudinal study of 263 youth (ages 2–16) with chronic physical illness and their parents, assessed at baseline (T1), six months (T2), 12 months (T3), and 24 months (T4). Parents reported on youth psychopathology using the Emotional Behavioural Scales (EBS). Latent profile analysis identified psychopathology profiles, and latent transition analysis quantified the probability that youth remained or moved between groups over time.

**Results:**

Four profiles were identified: low psychopathology (LP), primarily internalizing (PI), primarily externalizing (PE), and high psychopathology (HP). Homotypic continuity (i.e., remaining in the same profile over time) was lower for the PI, PE, and HP subgroups. Youth in the PI subgroup were more likely to transition to the LP, while those in HP showed greater stability, with many remaining in the high-symptom groups. Child age, parent psychopathology, and parent education significantly predicted profile transitions.

**Conclusions:**

Most youth showed changes in their mental health over time, but a small proportion with HP (<5%) had more persistent problems. Results demonstrate the need for early identification and intervention for youth at risk of chronic mental health difficulties.

## Plain Language Summary

**Why was the study done?:**

Youth with a chronic physical illness are more likely to experience mental health challenges, but their symptoms may change over time in different ways. Some youth may get better, while others may worsen over time. This study explored different mental health patterns in youth and how they shift over time to better understand ways to support their mental well-being.

**What did the researchers do?:**

The researchers studied different patterns of mental disorder symptoms in youth with chronic physical illness and how youth shift across different symptoms over a two-year period. They also explored if factors such as disability, parent psychological distress, and family income influence these changes.

**What did the researchers find?:**

Youth fell into four mental health profiles, including low, internalizing, externalizing, and high symptoms. Most youth remained in the same group over time. Youth with more severe disabilities were less likely to move into lower-symptom groups, suggesting persistent mental health challenges. Youth whose parents had lower psychological distress were also less likely to transition into the low or externalizing groups, highlighting a potential influence of family well-being.

**What do the findings mean?:**

Identifying mental health profiles and the stability and change across profiles over time can help clinicians tailor interventions to specific symptom patterns and address the dynamic nature of youth mental health.

## Introduction

Psychopathology is common among young people, with approximately one in five youth <18 years of age having one or more mental disorders involving symptoms of impairment (Georgiades et al., 2019). Risk factors such as family dysfunction/instability, parental psychopathology, and low socioeconomic status are associated with youth psychopathology (Fanti & Henrich, 2010; Fomby & Cherlin, 2007). The critical development stage from early childhood to late adolescence involves numerous biological changes (e.g., puberty), cognitive developments (e.g., identity formation), and socio-environmental transitions (e.g., moving to a new school). Struggles in adapting to these challenges increase risk of developing mental health problems (Göbel et al., 2022; Reitz et al., 2005). Indeed, most mental disorders have their onset during adolescence (Kessler et al., 2005) and affect outcomes over the life-course (Althoff et al., 2010; Caspi et al., 2020; Hofstra et al., 2002; Kessler et al., 2005; Waddell et al., 2018). Early onset of mental illness can result in a downward spiral of disadvantage and suffering among youth and their families, eventually leading to psychosocial, academic, and economic adversity (Malla et al., 2018; McLeod et al., 2012; Sallis et al., 2019; Sharpe et al., 2016).

Likewise, chronic physical illnesses-health problems requiring long-term management affect approximately 25% of youth, and its prevalence is increasing (Van Cleave et al., 2010). These children often experience mental health problems due to their physical illness or its treatment, leading to more severe symptoms and impairment in both physical and mental health (Merikangas et al., 2015; Van Der Lee et al., 2007). This dual burden, or multimorbidity, negatively impacts daily functioning, increases health service use, and increases the risk of suicide and substance abuse (Ferro et al., 2017; Luther et al., 2020; Qadeer et al., 2017, 2019; Reaume et al., 2021; Reaume & Ferro, 2019). About 40% of youth with chronic physical illness (CPI) screen positive for a mental disorder diagnosis, and this multimorbidity is persistent over time (Ferro et al., 2024; Tegethoff et al., 2015). Despite its prevalence and chronicity, research on physical-mental multimorbidity among youth is limited.

Given the burden of poor mental health, particularly among youth with CPI, examining the course of youth psychopathology can shed light on which aspects represent transient phases of development and which indicate persistent problems (Blok et al., 2022). Symptoms of psychopathology are typically categorized into two distinct groups: internalizing symptoms (e.g., depression) and externalizing symptoms (e.g., conduct disorder) (Sigurdson et al., 2015). Additionally, youth can have co-occurring and sequential comorbidities of internalizing and externalizing problems, and individuals can move between diagnoses over the lifespan (Healy et al., 2022). Multiple symptoms are linked to greater severity, persistence into adulthood, and more negative outcomes (Basten et al., 2013; Fanti & Henrich, 2010).

In early life, the risk of comorbidity increases due to the constantly shifting symptoms of psychopathology as children develop (McGorry et al., 2007; Wigman et al., 2013). These symptoms are often transient, typically decreasing with age due to the rapid developmental changes during childhood (Blok et al., 2022; Göbel et al., 2022). Some studies have tracked the fluidity of psychopathology throughout early development using approaches focused on symptoms and group-based trajectory modelling (Fanti & Henrich, 2010; Nivard et al., 2017; Olson et al., 2017). Yet, using a person-centred approach allows data to reflect that individuals may experience different symptoms at various times, enabling tracking of these symptom changes over time (Healy et al., 2022). This aligns with the observed patterns of simultaneous and sequential comorbidity (Caspi et al., 2020; Mcgrath, 2021; Plana-Ripoll et al., 2019) and the dynamic and unpredictable nature of psychopathology in youth (McGorry et al., 2007). Person-centred approaches assume that while there are groups of psychopathology symptoms within the population, individuals can shift between these groups over time (Healy et al., 2022). Many studies have used latent profile/class analysis to identify subgroups of youth with similar mental health symptoms, but few have examined a broad range of symptoms covering both the internalizing and externalizing spectrum (Blok et al., 2022; Healy et al., 2022; McElroy et al., 2017; Petersen et al., 2019). These studies typically find that the largest group comprises individuals with low symptom levels. Latent transition analysis tracks transitions between profiles, aligning with the dynamic nature of youth psychopathology. However, it has not been used in a sample of youth with CPI (Blok et al., 2022; Göbel et al., 2022; Healy et al., 2022; McElroy et al., 2017; McGorry et al., 2007; Spinhoven et al., 2012; Willner et al., 2016). Person-centred studies on youth can provide valuable insights into the early composition of psychiatric comorbidity and subsequent developmental trajectories (Kessler et al., 2007, p. 200). The present study aims to build on previous research by examining transitions in youth with a chronic physical illness—a subset of youth that has thus far not been the focus of the study. The interplay between chronic physical and mental illness underscores the need for a comprehensive approach to healthcare that addresses both the physical and mental health needs of youth. Understanding the stability and change in psychopathology in youth with chronic physical illness is crucial for developing targeted interventions that can mitigate these risks and improve overall health outcomes.

This study has three objectives: (i) to examine the latent structure of psychopathology in a sample of youth with a chronic physical illness, (ii) to examine person-level stability and change of these profiles over time, and (iii) to identify predictors of transitions over time. By examining individual-level transitions between internalizing, externalizing, and comorbid profiles over time, we may better understand how these profiles develop over time. To our knowledge, this study is novel in its application of latent transition analysis (LTA) to youth with CPIs, allowing us to model how multidimensional psychopathology profiles evolve, rather than treating them as static categories.

## Methods

### Design and sample

Data come from the Multimorbidity in Children and Youth Across the Life-course (MY LIFE) study (Ferro et al., 2019). This ongoing prospective study investigates the mental health of children with a CPI (e.g., asthma, diabetes, epilepsy). It includes 263 youth aged 2–16 years and their parents who attended outpatient clinics at McMaster Children’s Hospital (Hamilton, Ontario, Canada). The inclusion criteria required that youth have one physician-diagnosed CPI (defined as a condition expected to be existent for ≥ 12 months). Additionally, youth and parents were excluded if either could not understand English or if the child had >1 physical illness (i.e., medically complex) at the time of recruitment. For these analyses, follow-up data over 24 months were modelled with assessments at baseline (T1), six (T2), 12 (T3), and 24 (T4) months.

### Study procedures

Parents consented on behalf of children 2–6 years old, youth aged 7–15 years provided written informed assent, and all parents and youth ≥ 16 provided written informed consent. Data were collected using structured interviews and computer-assisted self-report questionnaires. Parent-reported data were collected for all participating youth and youth ≥ 10 years old provided self-reports.

### Measures

#### Youth psychopathology

Past six-month youth psychopathology was measured using the parent-reported 52-item Emotional Behavioural Scales (EBS) (Duncan et al., 2019). The EBS measures symptoms of internalizing [generalized anxiety (GAD), separation anxiety disorder (SAD), social phobia (SP), major depressive disorder (MDD)] and externalizing disorders [attention-deficit hyperactivity disorder (ADHD), oppositional defiant disorder (ODD), and conduct disorder (CD)] based on the Diagnostic and Statistical Manual of Mental Disorders (DSM-5-TR). Items on the EBS are rated as 0 = ‘never or not true,’ 1 = ‘sometimes or somewhat true,’ and 2 = ‘often or very true,’ where higher scores indicate greater psychopathology. The total score is calculated as the sum of all items. The EBS has demonstrated robust psychometric properties in clinical and general population samples of youth (Boyle et al., 2019; Duncan et al., 2019; Sajobi et al., 2017). Internal consistency was high in this sample ( α = 0.86).

#### Youth disability

The proxy-administered 12-item World Health Organization Disability Assessment Schedule (WHODAS) 2.0 was used to measure youth disability (Üstün et al., 2010). The WHODAS 2.0 assesses functioning across cognition, mobility, self-care, getting along, life activities, and participation. Parents reflected on the difficulty their child had with each item within the past 30 days using a five-point scale ranging from 1 (none) to 5 (extreme/cannot do). A composite score that summed all items, ranging from 12–60, was calculated with higher scores representing greater disability. The WHODAS 2.0 has demonstrated robust psychometric properties in this sample (Ferro et al., 2022) and the internal consistency was high ( α = 0.93).

#### Parent stress

The 18-item Parent Stress Scale (PSS) measures parent stress in four domains: rewards, stressors, loss of control, and satisfaction (Berry & Jones, 1995). Items are scored from 1 (strongly disagree) to 5 (strongly agree). The total score ranges from 18–90, with higher scores representing more parent stress. The scale has demonstrated reliability and validity in parents of children with CPI (Zelman & Ferro, 2018). Internal consistency was high in this sample (α = 0.86).

#### Parent psychological distress

The 20-item Center for Epidemiological Studies Depression (CES-D) Scale assessed parent level of depressive symptoms (Radloff, 1977). This scale evaluates negative and positive affect, somatic activity, and interpersonal relations over the past week. Parents reported how often they reported symptoms on a four-point Likert scale (0 = ‘rarely or none of the time’ [less than one day per week] to 3 = ‘most or almost all of the time’ [5–7 days per week]). Total scores range from 0 to 60, with higher scores indicating greater depressive symptomatology. The CES-D has strong psychometric properties in parents of youth with mental disorder (Dol et al., 2022) and it showed high internal consistency in this sample (α = 0.92).

The seven-item Generalized Anxiety Disorder scale (GAD-7) measures parents’ generalized anxiety symptoms (Spitzer et al., 2006). They indicated how many days in the past two weeks they experienced symptoms on a scale from 0 (not at all) to 3 (nearly every day). The score, ranging from 0 to 21, reflects the level of anxiety, with higher scores indicating greater anxiety. The GAD-7 also demonstrated high internal consistency in this sample (α = 0.88).

### Demographic characteristics

Parents reported information about youth and parent age, sex, and immigration status. Additionally, they were asked about their marital status (partnered relationship or not), highest level of education (completed a college/university degree or not), and household income (<$90,000 or ≥ $90,000). Demographic information is provided in Table 1 at each time point (T1-T4).

**Table 1.** Demographic characteristics.

	Baseline (T1)	6 months (T2)	12 months (T3)	24 months (T4)
Child				
Age (years), mean (SD)	9.5 (4.2)	10.0 (4.2)	10.5 (4.2)	11.5 (4.2)
Male, *n* (%)	138 (52.5)	—	—	—
Born in Canada, *n* (%)	248 (94.3)	—	—	—
Parent				
Age (years), mean (SD)	40.52 (6.5)	41.0 (6.5)	41.5 (6.5)	42.5 (6.5)
Female, *n* (%)	237 (90.1)	—	—	—
Born in Canada, *n* (%)	223 (84.8)	—	—	—
Partnered relationship, *n* (%)	228 (86.7)	222 (84.4)	213 (81.0)	202 (76.8)
College/university degree, *n* (%)	200 (76.1)	190 (72.2)	190 (72.2)	192 (73.0)
Household income ≥ $90,000, *n* (%)	156 (59.3)	153 (58.2)	147 (55.9)	153 (58.2)

### Statistical analyses

#### Latent profile analysis

Latent profile analysis (LPA) was applied to define psychopathology subgroups at T1, T2, T3 and T4, using scores from the EBS subscales as indicators (i.e., CD, ODD, ADHD, MDD, GAD, SA, SP). The log-likelihood, (AIC), Bayesian Information Criterion (BIC), and the sample-adjusted Bayesian Information Criterion (SABIC) were used to assess the fit of each model (Akaike, 1987; Schwarz, 1978; Sclove, 1987). Lower estimates of AIC, BIC, AND SABIC indicate better model fit (McElroy et al., 2017). Entropy of the models was evaluated with values closer to 1, indicating better classification, and all profiles obtained should have a minimum of 1% of the participants (Blok et al., 2022). Lastly, all profiles were inspected visually to ensure each additional profile had a distinct severity. Because 10 imputed datasets were used using multiple imputation, we could not ascertain the bootstrapped likelihood ratio test (BLRT) or Lo-Mednell-Rubin (LMR) test for each model. Profiles were defined by comparing mean scores in each of the profiles. Missing data were imputed under the assumption that data were missing at random (MAR) (Muthén & Muthén, 2017). Attrition in the MY LFIE study was minimal, with 30 subjects missing data by time point four (i.e., 24 months).

#### Latent transition analysis

Latent transition analysis (LTA) was used to calculate transition probabilities between psychopathology profiles. First, longitudinal measurement invariance using LTA was tested by computing two LTAs, one with and one without invariance constraints. Because this analysis used imputed data, we could not ascertain the information needed for the likelihood ratio difference test. Instead, the measurement invariant solution was used to facilitate the interpretation of profiles and corresponding transitions (Ryoo et al., 2018).

Next, we used the number of profiles based on the optimal model at each time point from the LPA to estimate the status prevalence and transition probabilities using LTA (Ryoo et al., 2018). Transition probabilities of profiles were estimated from T1 to T2, T2 to T3, and T3 to T4. Lastly, we tested predictors of transitions for each model in which demographic characteristics (i.e., child age, child disability, parent education, parent psychological distress, and household income) were included as covariates using multinomial logistic regression. These predictors were guided by the Andersen Model of Health Service use, which emphasizes the role of predisposing, enabling, and need-related factors in shaping health trajectories. Mental health service use, while an important factor (Reaume et al., 2021; Tegethoff et al., 2015), was not included in the current analysis as it is the subject of a separate investigation using this sample (Dol et al., 2025). Both LPA and LTA were conducted using MPlus v8.5 (Muthén & Muthén, 2017).

## Results

### Latent profile analysis

If two or more models fitted the data equally well, the optimal fit was based on visual inspection of the output and a preference for model parsimony. We could not discriminate between models at T1–T4 based on the loglikelihood, AIC, BIC, or SABIC because the estimates decreased with each increasing number of profiles added to the model. At T1, entropy determined that four or five profiles fit the data equally well. Visual inspection showed that a five-profile model would not have differed in severity from the profiles already identified in the four-profile fit. At T2, a four-profile was determined to be the best-fitting model as it had the highest entropy value. At T3, the three-profile fit had a higher entropy but decided to retain the four-profile fit because it had lower values for loglikelihood, AIC, BIC, and SABIC and was consistent with the previous two-time points. Lastly, at T4, a four-profile fit was determined to be the best-fitting model as it had the same entropy value as the three-profile fit, and the five-profile fit had <1% of participants in the smallest profile. Therefore, a four-profile model was determined to be the optimal model to use across all four time points for subsequent analyses. This optimal model was guided predominantly by entropy and prior research in youth, which identified four distinct profiles: low symptoms, internalizing, externalizing, and comorbid. Model fit indices are provided in Table 2.

**Table 2.** Model fit statistics for latent profile Models at T1, T2, T3, and T4.

	Number of profiles	LL	AIC	BIC	SABIC	Entropy	Smallest profile
T1	2	−4085.79	8215.59	8294.17	8224.42	0.91	25.48%
	3	−3978.30	8016.59	8123.76	8028.64	0.92	3.42%
	4	−3891.24	7858.47	7994.21	7873.73	**0.95**	1.90%
	5	−3834.35	7760.70	7925.02	7779.18	**0.96**	1.90%
T2	2	−4081.66	8207.32	8285.91	8216.16	0.96	14.83%
	3	−3991.06	7882.12	7989.28	7894.17	0.94	4.18%
	4	−3880.51	7737.02	7872.76	7752.28	**0.94**	2.66%
	5	−3777.47	7646.95	7811.27	7665.42	**0.95**	2.28%
T3	2	−3989.71	8023.41	8102/00	8032.25	0.94	20.15%
	3	−3882.71	7825.42	7932.59	7837.47	0.94	3.42%
	4	−3823.18	7722.35	7858.10	7737.62	**0.96**	3.04%
	5	−3768.84	7629.67	7793.99	7648.15	0.93	2.66%
T4	2	−3972.13	7988.26	8066.85	7997.10	0.92	20.15%
	3	−3830.18	7720.37	7827.53	7732.42	0.93	3.42%
	4	−3761.56	7599.11	7734.85	7614.37	**0.93**	3.42%
	5	−3686.28	7464.56	7628.88	7483.04	**0.95**	0.004%

AIC = Akaike information criterion, BIC = Bayesian information criterion, LL = loglikelihood, SABIC = Sample-Adjusted Bayesian information criterion.

**Figure 1.**
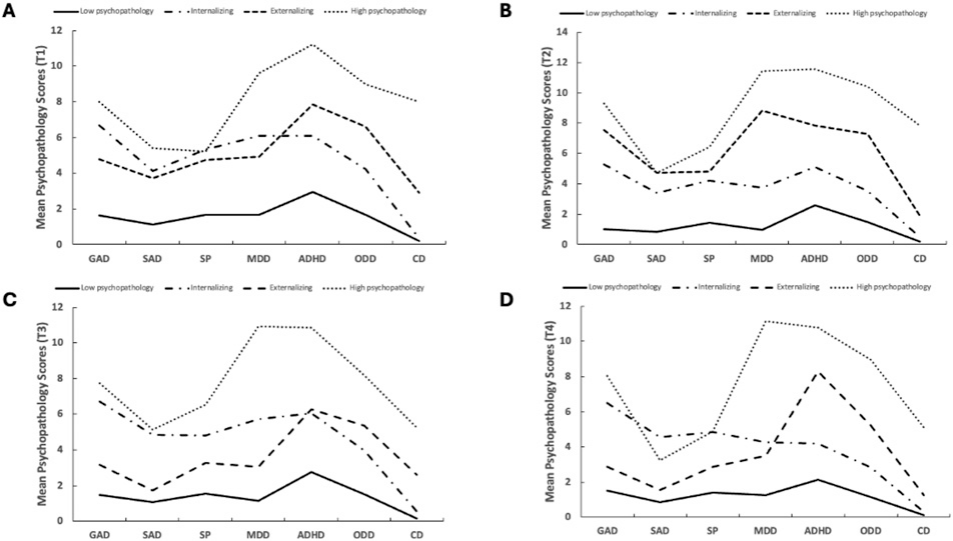
Psychopathology profiles, measured using the EBS, derived with latent profile analysis A at T1, B at T2, C at T3, and D at T4.

The four profiles are described as (1) low psychopathology (T1: 67.3%, T2: 62.7%, T3: 72.6%, T4: 68.1%) with low scores on all disorder subscales, (2) primarily internalizing (T1: 20.2%, T2: 27.0%, T3: 17.4%, T4: 15.2%), with higher scores on GAD, SA, SP, and MDD subscales (3) primarily externalizing (T1: 10.6%, T2: 8.0%, T3: 6.5%, T4: 12.9%) with higher scores on ADHD, ODD, and CD subscales, and (4) high psychopathology (T1: 1.9%, T2: 2.7%, T3: 3.0%, T4: 3.4%) with higher scores on all disorder subscales, see Figure 1.

### Latent transition analysis

Based on fit statistics, all three models for the LTA fitted the data equally well (T1 to T2: BIC = 15650.549, entropy = 0.93; T2 to T3: BIC = 15454.09, entropy = 0.94; T3 to T4: BIC = 15142.45, entropy = 0.95). Transition probabilities between T1-T4, based on invariant models, are shown in Table 3.

**Table 3.** Latent probability transition matrices of four-time points (Unconditional models).

Latent category	Time	Low psychopathology group	Primarily internalizing group	Primarily externalizing group	High psychopathology group
Low psychopathology group	**T1-T2**	**0.970 (145; 55.0%)**	0.030 (5; 1.7%)	0.000	0.000
	**T2-T3**	**0.963 (148; 56.4%)**	0.037 (6; 2.2%)	0.000	0.000
	**T3-T4**	**0.966 (159; 60.6%)**	0.013 (2; 0.8%)	0.021 (3; 1.3%)	0.000
Primarily internalizing group	**T1-T2**	0.237 (18; 7.0%)	**0.732 (56; 21.4%)**	0.030 (2; 0.9%)	0.001 (0; 0.0%)
	**T2-T3**	0.151 (12; 4.5%)	**0.767 (60; 23.0%)**	0.082 (7; 2.5%)	0.000
	**T3-T4**	0.241 (11; 4.1%)	**0.759 (34; 13.0%)**	0.000	0.000
Primarily externalizing group	**T1-T2**	0.000	0.339 (10; 3.9%)	**0.596 (18; 6.8%)**	0.065 (2; 0.8%)
	**T2-T3**	0.000	0.448 (9; 3.2%)	**0.552 (10; 4.0%)**	0.000
	**T3-T4**	0.089 (4; (1.5%)	0.013 (1; 0.2%)	**0.828 (36; 13.5%)**	0.070 (3; 1.1%)
High psychopathology group	**T1-T2**	0.142 (1; 0.4%)	0.000	0.000	**0.858 (6; 2.3%)**
	**T2-T3**	0.000	0.097 (1; 0.4%)	0.198 (2; 0.8%)	**0.705 (8; 3.0%)**
	**T3-T4**	0.000	0.114 (1; 0.4%)	0.276 (3; 1.1%)	**0.610 (6; 2.3%)**

1. Profiles on the x-axis represent T1, T2, and T3, respectively and profiles on the y-axis represent T2, T3, and T4, respectively.

2. Numbers represent transition probabilities (row sum = 100%).

3. *n* and % of each transition (out of 16) are shown in parenthesis.

Bold numbers indicate homotypic continuity.

Homotypic continuity, which indicates children classified in the same psychopathology subgroup at two consecutive time points, was 97.0% between T1 and T2 for the low psychopathology profile, 96.3% between T2 and T3, and 96.6% between T3 and T4. Homotypic continuity for primarily internalizing symptoms was 73.2% between T1 and T2, 76.7% for T2 to T3, and 75.9% for T3 to T4. For primarily externalizing symptoms, homotypic continuity was 59.6% for T1 to T2, 55.2% for T2 to T3, and 82.8% for T3 to T4. Lastly, homotypic continuity for the high psychopathology profile was 85.8% for T1 to T2, 70.5% for T2 to T3, and 61.0% for T3 to T4.

**Figure 2.**
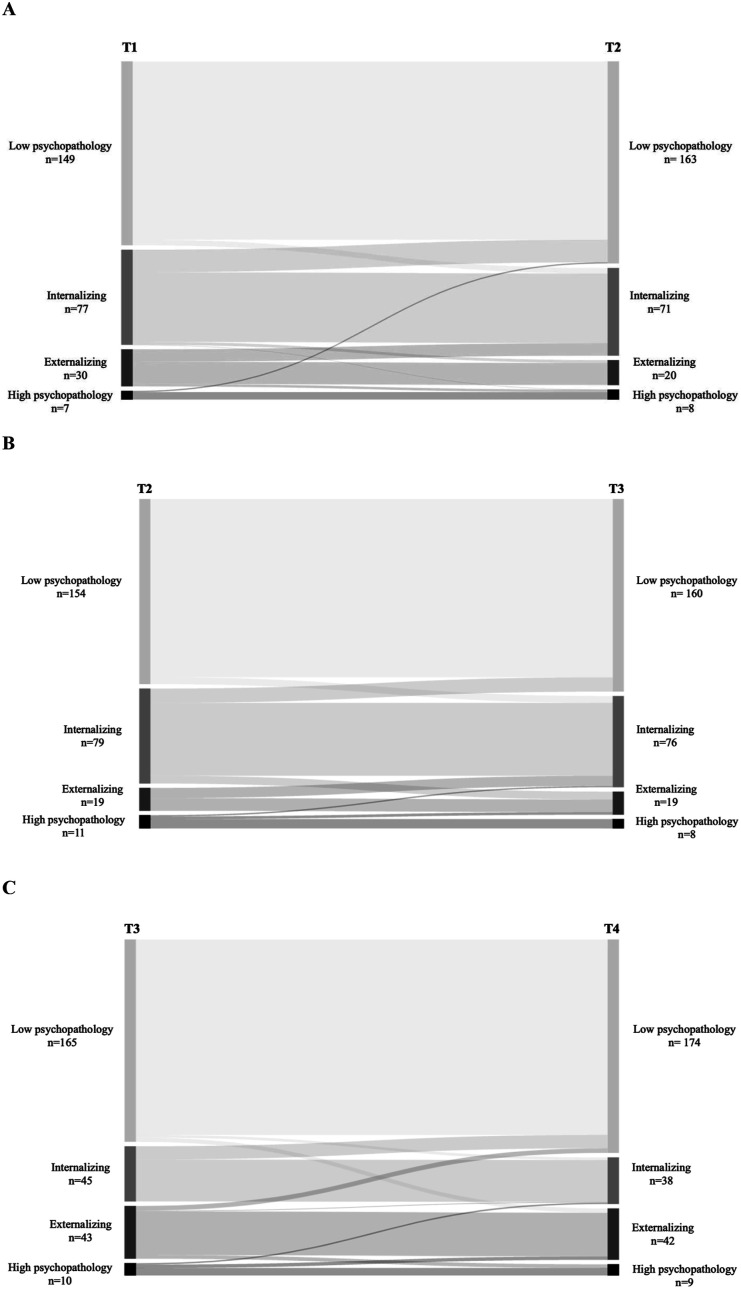
Sankey Diagram displaying transitions from A: T1-T2, B: T2-T3, and C: T3-T4 of the study.

Regarding heterotypic continuity, few children transitioned from the low psychopathology profile to the primarily internalizing (T1–T2: 3.0%; T2–T3: 3.7%; T3–T4: 1.3%) and primarily externalizing (T3–T4: 2.1%) profiles, but no children transitioned from low psychopathology into the high psychopathology profile. Few children transitioned out of the primarily internalizing profile into the primarily externalizing profile (T1–T2: 3.0%; T2–T3: 8.2%), whereas a greater number of children transitioned to the low psychopathology profile (T1–T2: 23.7%; T2–T3: 15.1%; T3–T4: 24.1%). No children transitioned from the internalizing profile to the high psychopathology profile. For the primarily externalizing profile, a larger number of children transitioned into the primarily internalizing profile (T1–T2: 33.9%; T2–T3: 44.8%; T3–T4: 1.3%), very few transitioned into the low psychopathology profile (T3–T4: 8.9%), and very few transitioned into the high psychopathology profile (T1–T2: 6.5%; T3–T4: 7.0%). Lastly, for the high psychopathology profile, some children transitioned into the low psychopathology (T1–T2: 14.2%) and primarily internalizing (T2–T3: 9.7%; T3–T4: 11.4%) profiles; however, more children transitioned into the primarily externalizing profile (T2–T3: 19.8%; T3–T4: 27.6%). No youth transitioned from the high psychopathology profile to the low psychopathology group between T2 to T3 and T3 to T4. Sankey diagrams that illustrate the transitions can be seen in Figure 2.

#### Predictors of latent transition probabilities

Table 4 displays the results of the conditional latent transition models. For T1-T2, compared to those who remained in the high psychopathology profile, youth with higher levels of disability had significantly lower odds of transitioning from the high psychopathology to low psychopathology profile at T2 [OR = 0.86 (0.78,0.95)]. Youth with higher levels of disability and those whose parents had lower levels of psychological distress had significantly lower odds of transitioning from the high psychopathology to the internalizing profile at T2 [OR = 0.76 (0.65,0.89); OR = 0.91 (0.86,0.97), respectively].

**Table 4.** Estimates for covariates predicting transition probabilities (*N* = 263).

Covariate	β (SE)	*p*	OR (95% CI)
**T1-T2**			
High psychopathology profile to low psychopathology profile versus remaining in the high psychopathology profile			
Child age	−0.12 (0.13)	0.329	0.89 (0.69, 1.14)
Child disability	**−0.15 (0.05)**	**0.002**	**0.86 (0.78, 0.95)**
Parent education	0.74 (0.77)	0.330	2.10 (0.46, 9.48)
Parent psychological distress	−0.03 (0.02)	0.238	0.97 (0.93, 1.01)
Household income	0.28 (1.00)	0.781	1.32 (0.19, 9.39)
High psychopathology profile to primarily internalizing profile versus remaining in the high psychopathology profile			
Child age	−0.15 (0.13)	0.224	0.86 (0.67, 1.11)
Child disability	**−0.27 (0.08)**	**0.001**	**0.76 (0.65, 0.89)**
Parent education	0.50 (0.83)	0.545	1.65 (0.32, 8.39)
Parent psychological distress	**−0.09 (0.03)**	**0.004**	**0.91 (0.86, 0.97)**
Household income	0.13 (1.02)	0.903	1.14 (0.15, 8.41)
High psychopathology profile to primarily externalizing profile versus remaining in the high psychopathology profile			
Child age	0.03 (0.12)	0.825	1.03 (0.81, 1.30)
Child disability	−0.05 (0.04)	0.266	0.95 (0.88, 1.03)
Parent education	0.42 (0.76)	0.579	1.52 (0.34, 6.75)
Parent psychological distress	0.02 (0.02)	0.394	1.02 (0.98, 1.06)
Household income	1.35 (1.05)	0.199	3.86 (0.49, 30.20)
**T2-T3**			
High psychopathology profile to low psychopathology profile versus remaining in the high psychopathology profile			
Child age	−0.19 (0.10)	0.044	0.83 (0.68, 1.01)
Child disability	**−0.12 (0.04)**	**0.001**	**0.89 (0.82, 0.96)**
Parent education	**1.54 (0.74)**	**0.037**	**4.66 (1.09, 19.89)**
Parent psychological distress	**−0.09 (0.02)**	**0.000**	**0.91 (0.88, 0.95)**
Household income	−0.76 (0.82)	0.351	0.47 (0.09, 2.33)
High psychopathology profile to primarily internalizing profile versus remaining in the high psychopathology profile			
Child age	**−0.33 (0.10)**	**0.001**	**0.72 (0.59, 0.87)**
Child disability	**−0.19 (0.05)**	**0.000**	**0.83 (0.75, 0.91)**
Parent education	1.63 (0.85)	0.056	5.10 (0.96, 27.00)
Parent psychological distress	**−0.09 (0.02)**	**0.000**	**0.91 (0.88, 0.95)**
Household income	−1.33 (0.89)	0.133	0.26 (0.05, 1.51)
High psychopathology profile to primarily externalizing profile versus remaining in the high psychopathology profile			
Child age	**−0.30 (0.11)**	**0.005**	**0.74 (0.60, 0.92)**
Child disability	**−0.44 (0.13)**	**0.001**	**0.64 (0.50, 0.83)**
Parent education	1.24 (0.79)	0.116	3.46 (0.73, 16.25)
Parent psychological distress	**−0.13 (0.03)**	**0.000**	**0.88 (0.83, 0.93)**
Household income	−2.07 (1.10)	0.061	0.13 (0.01, 1.09)
**T3-T4**			
High psychopathology profile to low psychopathology profile versus remaining in the high psychopathology profile			
Child age	−0.20 (0.10)	0.046	0.82 (0.67, 1.00)
Child disability	−0.09 (0.06)	0.108	0.91 (0.81, 1.03)
Parent education	−0.31 (0.80)	0.703	0.73 (0.15, 3.52)
Parent psychological distress	−0.02 (0.02)	0.477	0.98 (0.94, 1.02)
Household income	−0.12 (0.94)	0.895	0.89 (0.14, 5.60)
High psychopathology profile to primarily internalizing profile versus remaining in the high psychopathology profile			
Child age	−0.17 (0.10)	0.086	0.84 (0.69, 1.03)
Child disability	−0.01 (0.05)	0.928	0.99 (0.90, 1.09)
Parent education	0.84 (0.78)	0.284	2.32 (0.50, 10.68)
Parent psychological distress	0.02 (0.02)	0.459	1.02 (0.98, 1.06)
Household income	0.46 (0.95)	0.630	1.58 (0.25, 10.20)
High psychopathology profile to primarily externalizing profile versus remaining in the high psychopathology profile			
Child age	−0.19 (0.10)	0.048	0.83 (0.68, 1.01)
Child disability	**−0.27 (0.08)**	**0.001**	**0.76 (0.65, 0.89)**
Parent education	−0.05 (0.80)	0.950	0.95 (0.20, 4.56)
Parent psychological distress	**−0.05 (0.02)**	**0.019**	**0.95 (0.91, 0.99)**
Household income	−0.15 (0.89)	0.867	0.86 (0.15, 4.93)

CI = confidence interval; OR = odds ratio; SE = standard error.

For T2-T3, compared to those who remained in the high psychopathology profile, youth with higher levels of disability and those whose parents had lower levels of psychological distress had significantly lower odds of transitioning from the high psychopathology profile to the low psychopathology profile at T3 [OR = 0.89 (0.82,0.96); OR = 0.91 (0.88,0.95), respectively]. In contrast, those whose parents have a college/university degree had significantly higher odds of this transition [OR = 4.66 (1.09,19.89)]. Older youth, those with higher levels of disability, and those whose parents had lower levels of psychological distress had significantly lower odds of transitioning from the high psychopathology to the internalizing profile at T3 [OR = 0.72 (0.59,0.87); OR = 0.83(0.75,0.91); 0.91(0.88,0.95), respectively]. Older youth, those with higher levels of disability, and those whose parents had lower levels of psychological distress had significantly lower odds of transitioning from the high psychopathology to the externalizing profile at T3 [OR = 0.74 (0.60,0.92); OR = 0.64 (0.50,0.83); OR = 0.88 (0.83,0.93), respectively].

For T3-T4, compared to those who remained in the high psychopathology profile, youth with higher levels of disability and those who had parents with lower levels of psychological distress scores had significantly lower odds of transitioning from the high psychopathology to the externalizing profile at T4 [OR = 0.76 (0.65,0.89); OR = 0.95 (0.91,0.99), respectively].

## Discussion

This study aimed to identify transitions in psychopathology profiles in youth with chronic physical illness and the factors that are associated with transitions. Leveraging LTA revealed not only the prevalence of distinct symptom subgroups but also the probabilities of moving between them, offering nuanced insight into when and for whom integrated mental health interventions may be most beneficial. Similar to previous studies using LCA to investigate multiple psychopathology symptoms (Basten et al., 2013, 2016; Blok et al., 2022; Healy et al., 2022; McElroy et al., 2017), subgroups of psychopathology included four profiles: low psychopathology, primarily internalizing, primarily externalizing, and high psychopathology. It was observed that the stability of youth remaining within the high psychopathology profile decreases with age. Notably, while there is a change in symptoms for youth classified in the high psychopathology profile, all youth from this high-risk group from six to 12 months and 12–24 months remain in either the primarily internalizing, primarily externalizing, or high psychopathology subgroups over time.

The transition probability towards the low psychopathology profile decreased over time, whereas the probability of transitioning towards the internalizing profile remained stable, and transitioning to the primarily externalizing profile increased over time. Early interventions are, therefore, important to maximize the likelihood of positive outcomes. Like findings by Healey et al. (2022) in a population sample of youth between 12 to 24 months, most youth with CPI who moved into the high psychopathology subgroup had transitioned from the primarily externalizing profile. Thus, a small subset of these youth with primarily externalizing behaviours were at risk for worsening issues and might need ongoing monitoring (Healy et al., 2022). Continuous monitoring and early intervention could help prevent the escalation of psychopathology symptoms and reduce cross-domain comorbidity. In line with McElroy et al. (2017), youth with primarily internalizing symptoms were more likely to transition to the low psychopathology profile over time compared to those with primarily externalizing problems, indicating that internalizing symptoms specifically are a transient phase of development for many youths. Additionally, few youths transitioned from primarily externalizing to the low psychopathology profile, suggesting that externalizing psychopathology may confer a higher risk of developing cross-domain comorbidity compared to internalizing psychopathology (McElroy et al., 2017). Consequently, identifying risk factors in youth with externalizing problems, worsening trends, and intervening early may help to lower the risk of comorbidities, societal health burdens, and social costs (Healy et al., 2022).

As expected, the largest number of youths were classified in the low psychopathology profile. Homotypic continuity was high for all three transition groups. This suggests that most youth who exhibit low levels of psychopathology early on will continue to have low symptom levels as they age. Only 2–4% of those with initially low symptoms shifted to the primarily internalizing profile over time, and 1% moved to the primarily externalizing profile. Compared to previous work (Blok et al., 2022, 2022, 2022), this study found higher homotypic continuity for all subgroups. The homotypic continuity might be high because the follow-up period is only two years, which may not be long enough for symptoms to change significantly across subgroups. Future studies should examine transitions in youth with CPI over longer follow-ups to better understand how psychopathology symptoms evolve, which may inform the timing and nature of interventions. This supports the implementation of early screening to identify youth at low risk and provide ongoing support for more severe symptoms, ensuring appropriate allocation of resources for long-term mental health outcomes.

Findings from Figure 2 show that between baseline and six months and six to 12 months, the proportion of children with primarily internalizing symptoms remains stable, whereas there is a notable decrease in the proportion of children classified in the primarily internalizing group at 12–24 months. The stable prevalence observed from baseline to 12 months indicates a certain stability in the prevalence of internalizing symptoms, with more youth reporting mainly internalizing symptoms during this period. This is contrary to previous research, which suggests that the risk of internalizing problems increases with age (Lippold et al., 2021). Potential reasons for this inconsistency could include differences in the age range of the study participants, variations in the assessment tools used to measure internalizing symptoms or unique characteristics of the study populations that influence the trajectory of internalizing problems over time. Kessler et al. (2005) also describe that anxiety disorders can emerge at any age, whereas mood disorders are more likely to appear during adolescence. While internalizing symptoms may be stable initially, they may decrease over time, indicating a possible widow for early intervention. Future research should explore factors influencing internalizing symptoms over time, including age-specific risks and measurement methods.

On the other hand, results showed a decline in children classified in the primarily externalizing profile between baseline and six months and six to 12 months, followed by a notable increase in children classified in the primarily externalizing group at 12–24 months. This aligns with earlier studies using LPA (Blok et al., 2022; McElroy et al., 2017) and the understanding that externalizing disorders can develop into late childhood (Keenan et al., 2010; Kessler et al., 2005; Maughan et al., 2004). However, other longitudinal research has shown that the prevalence of externalizing disorders decreases from childhood to adolescence (Costello et al., 2011). These findings imply that youth transitioning to the primarily externalizing profile were mainly those in the high psychopathology group at earlier time points (Blok et al., 2022; McElroy et al., 2017). Moreover, those youth who transitioned out of the primarily externalizing profile transitioned mainly to the primarily internalizing profile between baseline and six months and six to 12 months and to the low psychopathology group from 12 to 24 months. There is a need to explore the pathways of externalizing behaviours and their interactions with other psychopathology profiles. Additionally, programming should focus on early risk factors for externalizing disorders to prevent long-term behavioural problems in youth with CPI.

The prevalence of high psychopathology was relatively stable across time in this study. In contrast, previous studies found that the prevalence of the high psychopathology profile is highest in late childhood, after which there is a decline into adolescence (Blok et al., 2022; Deutz et al., 2018). This could be due to a relatively small sample size compared to other studies that used large population-based samples. However, our results support the evidence that the high psychopathology subgroup is an at-risk state for persistent psychopathology (Blok et al., 2022; Healy et al., 2022). Where between baseline and six months, only 14% of youth in the high psychopathology profile transitioned to the low psychopathology profile, and no youth transitioned to the low psychopathology profile between six to 12 months and 12–24 months. Studies need to explore different factors that contribute to the persistence of high psychopathology.

Regarding predictors of transitions, the analysis suggests that youth with more severe disabilities were consistently less likely to transition out of the high psychopathology group into less severe psychopathology profiles over time. Specifically, youth with worse disability and those whose parents had better psychological distress scores were less likely to move into the low psychopathology or externalizing groups at different intervals, including from baseline to six months and six to 24 months. Additionally, older youth and those with greater disability were less likely to transition into the internalizing group between 6 and 12 months. Given the influence of youth disability and parent psychopathology on youth transitions over time, efforts are needed to support youth with higher levels of disability as they are less likely to transition out of high-risk psychopathology over time. For practice, this highlights the importance of early, comprehensive care that includes tailored interventions for youth with disability. Future research should identify specific factors that can facilitate positive transitions in this population.

This study should be considered in the context of its limitations. First, youth psychopathology was parent-reported due to the limited number of age-eligible youth available to provide self-reports. Parent informants frequently exhibit a negative bias and tend to have low agreement with youth informants (Oltean & Ferro, 2019). Second, the observational design limits causal inference, and although latent transition analysis offers insight into longitudinal patterns, findings should be interpreted as descriptive rather than predictive. Third, our clinical sample was relatively small and homogenous regarding family composition, socioeconomic status, and ethnicity which may impact the generalizability of our findings. Additionally, some potentially important predictors – such as peer relationships and family-level stressors—were not measured. Future research with more diverse samples and a broader range of exposures is needed to better understand individual risk trajectories and the mechanisms underlying change. Fourth, although we were unable to determine the proportion of our sample relative to the total population served by McMaster Children’s Hospital, the sociodemographic and clinical characteristics of the MY LIFE cohort have been shown to align with epidemiological data on a representative sample of Ontario Children with CPI (Reaume et al., 2024). Fifth, our study included a wide age range that spanned multiple developmental periods; thus, we could not ascertain how youth transitioned across subgroups across these developmental periods. Sixth, we did not have access to medical records or information on services received between time points; we could not account for the impact of access to mental health services.

## Conclusion

This study examined the prevalence and characteristics of carious psychopathology subgroups and changes in symptoms over two years. Four behavioural profiles were identified: low psychopathology, primarily internalizing, primarily externalizing, and high psychopathology. While many youths showed transitions between these profiles, those in the high psychopathology profile were more likely to have persistent symptoms. All youth in the high psychopathology profile between 6 and 24 months remained in a high-risk state. These findings suggest that internalizing and externalizing symptoms may be transient, whereas high psychopathology indicates a greater risk for long-term mental health issues. There is a need for more integrated services that address both mental health and disability to improve long-term outcomes for youth with CPI.

## Data Availability

The authors will not make their data and study materials available to other researchers.*
